# Should one trust experts?

**DOI:** 10.1007/s11229-021-03203-7

**Published:** 2021-05-21

**Authors:** Hein Duijf

**Affiliations:** Department of Philosophy, Vrije Universiteit Amsterdam, De Boelelaan 1105, 1081 HV Amsterdam, The Netherlands

**Keywords:** Trust, Experts, Social epistemology, Interest alignment, Competence

## Abstract

Should one trust experts? My answer to this question is a qualified ‘no’ (and a qualified ‘yes’). In this paper I explore the conditions under which it is rational to trust and defer to experts, and those under which it may be rational to refrain from doing so. I draw on two important factors for an actor’s trust in a partner: trust depends on the partner’s competence and on the partner’s interests (and benevolence). I propose that the conditions under which it is rational to trust and defer to experts depend on *the competences of the layperson and the expert,* and *the degree of interest alignment*. I present a model that demonstrates that it can be practically infeasible and even logically impossible to determine the expert’s level of competence and the degree of interest alignment. Although it may sound pessimistic that one can rationally refrain from trusting experts, I will also explore some more optimistic conclusions.

## Introduction

It is a platitude that ordinary citizens are well advised to trust and defer to experts in questions concerning the underlying causes of climate change, the risks of vaccination hesitancy, and the prospects of economic policy proposals. The reasoning is that in virtue of the experts’ expertise and competence on a given matter, citizens ought to defer—especially under the assumption that citizens could not possibly know any better than experts. This paper questions the validity of this reasoning and the correctness of the associated widespread belief that citizens ought to always defer to experts.

The aforementioned reasoning neglects the importance of the experts’ interests or motivations. For example, vaccination hesitancy increased in the last decades, in particular, due to the so-called “vaccination myths” that vaccination is linked to the onset of autism. These vaccination myths are hard to dispel using scientific arguments (Horne et al., 2015) or other fact-based communication strategies and interventions (Nyhan et al., 2014). Instead, (the perception of) conflicting interests and mistrust towards ‘Big Pharma’ play a crucial role in persistent vaccination hesitancy.^1^

Over and above rebutting the correctness of the widespread belief that it is rational to trust experts, I argue that the belief is questionable exactly in cases where there is insufficient alignment of interests between the layperson and the expert.^2^ (To clarify, in these cases, it is permissible for the layperson not to trust the expert, as opposed to the layperson being obliged to distrust the expert.) Indeed, the layperson may very well know that the expert is more competent and more knowledgeable in a given domain, yet *rationally* decide against deferring to the expert’s opinion in case their interests or values conflict.^3^

My analysis is based on a simple model of trust which draws on the common idea that trust involves two dimensions: competence and motivations.^4^ On the one hand, a student trusts their math teacher to show them the right outcome of some calculation because the teacher is very reliable in doing so. One would say that the math teacher is competent in the area of calculation. It may be that the degree of competence required for trust varies, but some competence is surely needed. On the other hand, the possibility that someone can betray one’s trust means that trusting someone else leaves open the possibility of betrayal.^5^ This entails that one should only trust someone else if the other is (or, is perceived to be) sufficiently benevolent. True enough, it may be that the degree of benevolence required for trust varies between contexts, but motivations play a crucial role.

In epistemology, trust and expertise have traditionally sat uneasily with individual epistemic autonomy (Adler, 2017; Lackey & Sosa, 2006). However, there is a consensus that much of our knowledge relies on trust (Coady, 1992; Hardwig, 1991) and that there are many occasions where we ought to defer to experts (Anderson, 2011; Hardwig, 1985).^6^ Almost everyone agrees that the trustworthiness of experts depends on their epistemic competence, moral character and social responsibility (Anderson, 2011; Goldman, 2001; Hardwig, 1991). Some philosophers have emphasized and studied the social factors and institutions that influence assessments of experts’ trustworthiness and credibility (Fricker, 1998; Rolin, 2020).^7^ Although this social dimension may play a crucial role, my analysis focuses on the role of epistemic competence and interests of experts in determining whether a layperson should trust and defer to them.

One may wonder about the distinction between two types of epistemic trust: i.e. between trusting the purely factual claims of experts as opposed to trusting the expert’s practical advice.^8^ Leaving aside questions about the nature and validity of this distinction, I am confident that my model can be used to study both species of epistemic trust. The extent to which laypeople should trust and defer to experts on purely factual matters ultimately depends on the degree of interest alignment. Often times, social epistemologists assume that the main goal of epistemic exchanges is to promote true beliefs and avoid errors (or some other purely epistemic goal). Although I doubt that experts and laypeople are always motivated in this way, my model is compatible with this assumption and can, hence, also be used to study the exact conditions under which laypeople should trust and defer to experts on purely factual matters.

One of the key innovations of my analysis is to operationalize the motivational dimension by considering *degrees of interest alignment*, as opposed to the binary distinction between conflict and mutuality. Let me provide some examples to illustrate why such a scale is important and to highlight different reasons why interests may (mis)align only partially.^9^ First, consider the case where a patient needs to decide whether to trust the general practitioner on multiple occasions. In such cases, it may be that the interests of the medical expert and the patient align on one occasion and conflict in another. A scale could then be thought of as the likelihood that their interests align. Second, consider the case where a political expert advises a layperson on which political party to vote for. Although the political expert may be more knowledgeable on the standpoints of various political parties on a wide variety of topics, it may well be that the expert does not know the interests and values of the layperson. As a result, the expert’s advice may be based on interests that differ from those of the layperson. The degree of interest alignment could then be thought of as the likelihood that the expert accurately determines the layperson’s interests. Third, a policy expert may advise laypeople on which rules to conform to. In these cases, it is plausible that the policy expert’s advice is aimed at serving the common interest irrespective of whether these fully align with the personal interests of the layperson.^10^ Fourth, a scientist may inform the public that there is no causal link between the MMR vaccine and autism.^11^ One’s confidence in this factual claim depends on judgements of inductive risk, which are partly influenced by one’s interests. It seems plausible that the scientist’s main concern is public health and a parent’s primary concern is her child’s health. Therefore, the consequences of false negatives may be different for the scientist and the parent. When we think of the risk-related interests of the scientist and the parent as levels of statistical significance, then these will typically only partially overlap.

In addition to motivating that the alignment of interests may come in degrees, these four examples help disambiguate malignance and conflicting interests. First, they demonstrate that malignance and conflicting interests are distinct: conflicting interests may even obtain for benevolent experts. Second, they illustrate that conflicting interests could arise either because the expert tries and fails to identify the interests of the layperson or because the expert is not responsive to the layperson’s interests. Third, conversely, they show the possibility that the interests fully align while the expert does not try to identify the interests of the layperson.

Although my critique of the widespread belief that it is always rational to trust experts may support a very bleak picture where laypeople can rationally resist and defy scientific knowledge, I will argue that there is also a positive side. In fact, my analysis allows to specify the conditions under which deference to experts is rational or advisable. For instance, it is important to note that deference to experts can be advisable *even if* the interests of the layperson and expert do not completely match.^12^ As I will argue below, depending on the exact level of discrepancy between the competences of the layperson and the expert, relatively low degrees of mutual interest could already support the rationality of deference to experts. The upshot is that mitigating distrust in science may only require a wide belief that scientists share at least some of the interests of the citizens.

Four clarifications are in order. First, my focus on the interests of laypeople and experts does not assume that they are self-interested or egoistic. These interests should be understood as covering any motivationally relevant factor including, but not limited to: self-interest, altruism, equality, fairness, reputation, common interests, corporate interests, and governmental interests. Second, I do not develop an explicit account of expertise: one main benefit of my analysis is that it applies to any account that assumes that experts are more knowledgeable than laypeople (in a given domain). Third, in light of recent philosophical work on trust in science (Irzik & Kurtulmus, 2019; Wilholt, 2013), it may be helpful to emphasize that my analysis does not focus on the realm of science: it applies to any expert, at least insofar as the expert is more knowledgeable in a given domain than the layperson.

Lastly, one might think that trust in experts requires a high degree of interest alignment.^13^ For example, a knowledgeable investor might tell a layperson to invest in a multinational oil corporation. However, if the layperson wishes to have high expected returns, but also wants to avoid investing in environmentally damaging industries, one might defend the claim that the knowledgeable investor is not highly expert *in relation to* this layperson. Let me emphasize, once more, that low degrees of interest alignment may even obtain for benevolent and responsible experts.^14^ Moreover, irrespective of the general validity of this claim, my model can be used to study these specific cases. After all, one could focus the analysis on the circumstances where experts have high degrees of interest alignment and, then, study whether laypeople should trust and defer to experts. In these circumstances, my analysis illustrates that the extent to which laypeople should trust and defer to experts depends on their respective competences and the degree of interest alignment. The strength of the model lies precisely in the fact that it can accommodate different sets of assumptions.

The rest of the paper will, for simplicity’s sake, concern cases where a layperson must decide between two alternatives and where one of these alternatives is correct or serves her interests best. We assume that both the expert and the layperson form their own opinion on which alternative to choose and, subsequently, the expert communicates her advice to the layperson. The layperson then bases her final choice on her own initial opinion and on the expert’s advice.

In what follows, I assume that the question of whether the layperson ought to defer to the expert depends on whether she thinks it is more likely that the expert identifies the alternative that best serves the layperson’s interests. One might call this the argument from *accuracy*. The choice of whether to defer to experts is most prominent in cases of disagreement and it depends on the likelihood of (a) the expert advice being correct versus that of (b) the layperson’s initial opinion being correct. Note that this notion of correctness concerns whether the identified alternative best serves the layperson’s interests, as opposed to an absolute notion of correctness. I use this non-absolute notion of correctness throughout the paper.

To apply my model more generally to study trust and distrust, it is important to also consider asynchronous aspects of trust. That is, relationships of trust and distrust often develop over a period of time and over several interactions. From this perspective, the present relationships of trust and distrust are partly the result of past exchanges and experiences. To make a start with studying the dynamics of trust in experts, I distinguish between cases depending on whether the layperson is able to find out which alternative best suited her interests later. First, under the simplified assumption that the layperson later learns about what the correct alternative was, we can postulate the following dynamics of trust. On the one hand, the layperson would increase her trust in the expert upon learning that the expert was correct. It seems plausible that this positive effect is greater when the layperson disagreed rather than agreed beforehand. On the other hand, the layperson would decrease her trust in the expert when she learns that the expert’s advice did not pick out the option that best serves the layperson’s interests. This negative effect is plausibly greater when they disagreed beforehand (implying that the layperson was correct). I will therefore discuss how the two-dimensional model of trust (involving competences and interests) can be used to study the following events: (I) the event where the expert is incorrect (irrespective of the layperson’s opinion), and (R) the event where the layperson would regret having deferred to the expert, i.e. where the layperson was correct while the expert was incorrect.^15^

Second, in the absence of the possibility to later learn what the correct alternative was, it is plausible that higher rates of disagreement yield lower degrees of trust. I therefore also consider (D) the event where the layperson and expert disagree.

It is therefore important to distinguish between several possible events: the expert’s advice correctly identified the alternative that best serves the layperson’s interests or not; and the expert and layperson agree or disagree. These possibilities are summarized in Table 1.

**Table 1:** Different cases

	Agreement	Disagreement
Correct expert advice		
Incorrect expert advice		

My simple model will be used to study these asynchronous aspects of the dynamics of trust and to argue that it can be logically impossible to determine the expert’s competence and the degree of interest alignment. Moreover, even if the expert’s competence is known, the model demonstrates that it can be practically infeasible to determine the exact degree of interest alignment.

The paper is structured as follows. I start in Sect. 2 by outlining the basic model of trust that emphasizes the role of competence and interest alignment by considering the two extreme cases of mutual interests and conflicting interests. I extend the model in Sect. 3 to cover intermediate degrees of interest alignment, to analyse the three previously mentioned events, and to present the exact conditions under which it is rational to trust and defer to experts. In Sect. 4, I use the model of trust to study the revision of trust and the question of whether it is logically or practically feasible to determine the exact level of competence and degree of interest alignment. In Sect. 5, I explore and highlight some optimistic ramifications.

## The model and its predictions

To simplify matters, let us imagine that the layperson is to make up her mind on a certain question, let us say whether Q is the case. Let us suppose that the layperson is only mildly competent in finding out whether Q is the case on her own. The layperson’s individual competence is modelled by a probability L, where 0.5 ≤ L ≤ 1, and assume that L is close to 0.5.^16^ The layperson’s competence L can be taken to represent the chance that she makes up her mind correctly regarding Q, when left to her own devices. The assumption that 0.5 ≤ L means her competence is not worse than mere chance level.^17^ In contrast, the expert’s competence exceeds that of the layperson. The expert’s competence is modelled by a probability E, where 0.5 ≤ E ≤ 1. Since experts are more knowledgeable than laypeople, I assume that E exceeds L. Once again, the expert’s competence E can be taken to represent the chance that she makes up her mind correctly regarding Q. This simplified model assumes that the chances associated with the expert and the layperson are probabilistically independent. For instance, this means that the chance that the layperson makes up her mind correctly regarding Q is independent of whether the expert does so, and vice versa.

In this simple model, it should be obvious that the expert is more knowledgeable than the layperson. The model explains this by postulating that the expert has a higher chance of correctly making up her mind regarding Q.

A basic extension of this simple model can be used to prove the main claim of this paper: it can be rational to refrain from deferring to experts. The underlying idea is that it may be hard to disentangle our epistemic lives from our practical ends. Let us imagine a scenario where there is a question whether Q and this determines the policy that best suits one’s interest. For simplicity’s sake, let us imagine that if Q is true, then it is in the layperson’s interest to choose alternative A_1_ and if Q is false, then it is in her interest to choose alternative A_0_ instead.^18^

We will start by investigating the model’s predictions for the two simple cases where the expert and the layperson have mutual interests (Sect. 2.1) and where the expert and the layperson have conflicting interests (Sect. 2.2). Then, we will consider the more complex case where the interest alignment of the expert and the layperson lies somewhere in between mutuality and conflict (Sect. 3).

Before diving into the model’s predictions, I would like to add a clarification on the role of this model in my philosophical analysis. The model is formulated primarily for illustrative purposes and, most importantly, to indicate and give more substance to the argument against the widespread belief that it is (always) rational to defer to experts. I am confident that several idealizations could be dropped while preserving the main result that this widespread belief is not universally true. For example, although one may question the independence assumption of the model, I am confident that the main result remains true even in cases where the chances associated with the expert’s competence and the layperson’s competence are partially probabilistically dependent. Moreover, I am mostly interested in the qualitative predictions of the model as opposed to the quantitative predictions.

### Mutual interests

To start probing the idea that rational trust also depends on motivations or interests, let us assume that the expert’s interests *align* with those of the layperson. This means that if Q were true, then alternative A_1_ is also in the expert’s interest and if Q is false, then alternative A_0_ is in her interest. Under these assumptions, it should be obvious that the expert has a higher chance of getting it right. That is, the layperson has a higher expected chance of success if she trusts and defers to the expert.

In line with our previous observations, there are three interesting events that deserve attention. The first is the event where the layperson and the expert disagree (notation: D). The likelihood of this event depends on the competence of the layperson (L) and the competence of the expert (E). The likelihood of disagreement (notation: P(D)) is given by the sum of (a) the likelihood that the layperson is correct and the expert is incorrect (L · (1 − E)) and (b) the likelihood that the layperson is incorrect and the expert is correct ((1 − L) · E). Hence:

$$P\left( D \right) = L \cdot \left( {1 - E} \right) + \left( {1 - L} \right) \cdot E.$$

We may wonder how the likelihood of disagreement will change when we unilaterally change one of these variables. To answer this question, consider the following derivatives:

$$\frac{\partial P\left( D \right)}{{\partial L}} = 1 - 2E,$$

$$\frac{\partial P\left( D \right)}{{\partial E}} = 1 - 2L.$$

Given our assumption that L ≥ 0.5 and E ≥ 0.5, we see that (1 − 2E) ≤ 0 and (1 − 2L) ≤ 0. Hence, these derivatives reveal that the likelihood of disagreement decreases both when the competence of the layperson increases and when the competence of the expert increases (which should come as no surprise). So, under these circumstances, one way to prevent disagreement is by increasing competence of any of the two agents (or both).

The simple model can be used to approximate the magnitude of the risk of disagreement. These derivatives illustrate that the likelihood of disagreement decreases if the competence of the layperson or the expert increases. This means that the highest risk of disagreement occurs when L = E = 0.5, in which case the likelihood of disagreement is 50%. As a result, in all other cases the chance of disagreement is less than or equal to 50%. For example, when L = E = 0.8, the likelihood of disagreement is 32%. Hence, the model predicts that the risk of disagreement is highest in cases where the layperson and expert have the lowest competence.

The second event is where the expert’s advice picks out the incorrect alternative (notation: I), regardless of whether they agree on which alternative to choose. (Recall that being correct means that the person identifies the alternative that is in the layperson’s best interest.) The likelihood of this event (notation: P(I)) is given by the likelihood that the expert is incorrect:

$$P\left( I \right) = 1 - E.$$

It should be obvious that the likelihood of the expert’s incorrectness decreases as her competence increases. So, under these circumstances, the obvious way for the expert to prevent such errors is by increasing her competence.

We pay special attention to the event where the layperson would regret trusting the expert. That is, the event where the layperson and the expert disagree, the layperson correctly identifies the policy that is in the layperson’s interest, and the expert’s advice picks out the incorrect alternative. This phenomenon is important for the concept of trust and especially for the dynamics of trust. In virtually all cases, people are not certain about the competence and motivations of experts. So, it is not hard to imagine that such an event would cause and justify people to lower their trust in experts. It could be that people will no longer defer to experts in the future. Hence, there may be a justifiable negative feedback loop on trust, leading to distrust. Let us denote this event by R (which is meant to refer to Regretting trusting the expert).

In these circumstances, the likelihood of regret (notation: P(R)) is given by the likelihood that the layperson is correct while the expert is incorrect:

$$P\left( R \right) = P\left( {I\& D} \right) = L \cdot \left( {1 - E} \right).$$

It may be helpful to note that these equations illustrate that the likelihood of this event depends on the competence of the layperson, L, and the competence of the expert, E.

It follows immediately that the likelihood of this event can be decreased by (a) increasing the competence of the experts or by (b) decreasing the competence of the layperson. So, although the model exemplifies the possibility of a negative feedback loop on trust (even in cases of mutual interests), it also allows us to quantify the likelihood of such an event. The risk of negative feedback loops that are based on these events where the layperson regrets trusting the expert is greater when the difference between the layperson’s competence and that of the expert is smaller. As a consequence, in a society of knowledgeable citizens it is more likely that the citizens will regret deferring to experts than in a society of ignorant citizens, and, therefore, the likelihood of such negative feedback loops on trust also increases.

It may, once again, be helpful to explore the severity of this risk. Under the assumptions that 0.5 ≤ L ≤ E, it can easily be verified that the maximum risk is when L = E = 0.5, in which case the likelihood of this event is 25%. This means that, in general, the likelihood of this event is less than or equal to 25%.

The probabilities of the four previously mentioned events are given in Table 2.

**Table 2:** Mutuality: probabilities of events

	Agreement	Disagreement
Correct expert advice	L · E	(1 − L) · E
Incorrect expert advice	(1 − L) · (1 − E)	L · (1 − E)

### Conflicting interests

Now, let us proceed to the case where the interests of the expert *conflict* with those of the layperson. That is, if Q is true, then alternative A_0_ is in the expert’s interest and if Q is false, then A_1_ is in her interest, whereas the opposite is the case for the layperson. Under these circumstances, I will show that the layperson should not trust and defer to the expert.

We are again interested in the two outcomes discussed previously, denoted by D and I. First, what are the odds that the expert and the layperson disagree on which policy proposal to adopt? Each person’s judgment is based on that person’s competence and interests. The likelihood of disagreement (P(D)) is given by the sum of (a) the likelihood that both the layperson and the expert identify the alternative that is in their own respective interest (L · E) and (b) the likelihood that both fail to identify the alternative that is in their own respective interest ((1 − L) · (1 − E)). Hence:

$$P\left( D \right) = L \cdot E + \left( {1 - L} \right) \cdot \left( {1 - E} \right).$$

This equation illustrates that the likelihood of this event depends on the competence of the layperson (L) and the competence of the expert (E). To investigate how the likelihood of disagreement will change if we alter one of these two variables, consider the following derivatives:

$$\frac{\partial P\left( D \right)}{{\partial L}} = 2E - 1,$$

$$\frac{\partial P\left( D \right)}{{\partial E}} = 2L - 1.$$

Given the assumption that L ≥ 0.5 and E ≥ 0.5, we see that (2E − 1) ≥ 0 and (2L − 1) ≥ 0. Hence, these derivatives reveal that the likelihood of disagreement increases when the competence of the layperson and/or of the expert increases. So, the model predicts that one way to prevent disagreement is by decreasing competence of any of the agents (or both). Notice that this is exactly opposite to the previous case in Sect. 2.1, because the expert’s interests differ.

This reveals a benefit for authoritarian governments to keep citizens in the dark. After all, one way to prevent disagreement between a competent elite and a less competent proletariat with conflicting interests is to decrease the competence of the proletariat. In these cases, the likelihood of disagreement has been limited so that the layperson may agree often enough to support an elite with conflicting interests.

How severe is the risk of disagreement? It can be easily verified that the chance of disagreement varies between 0.5 and 1, where the lowest chance of disagreement is associated with L = E = 0.5.

The second event is where the expert picks out the incorrect alternative, irrespective of whether they agree on which alternative to choose (denoted by I). The probability of this event is given by the likelihood that the expert identifies the alternative that is in her own interest:

$$P\left( I \right) = E.$$

It immediately follows that, under the assumption of conflicting interests, the layperson should not trust and defer to the expert. After all, the probability that the expert picks out the correct alternative equals 1 − E, which does not exceed 50%, while the probability that the layperson does so equals L, which is assumed to exceed 50%.

The third event of interest is where the layperson regrets having trusted the experts (denoted by R). It is especially important in circumstances where laypeople later find out which policy proposal is in their own respective interest. As previously noted, this outcome is especially important for the dynamics of trust since it may trigger a negative feedback loop on trust. That is, we focus on the outcome where a given policy P is in the interests of the layperson, the layperson correctly judges that P best suits her interests, and the expert advises against P. Of course, in reality it may be hard to find out which policy proposal actually best suits one’s interest. Nonetheless, because the interests of the layperson and expert conflict, the probability of regret (P(R)) is given by the likelihood that both succeed in identifying the alternative that is in their own respective interest:

$$P\left( R \right) = L \cdot E.$$

As a result, it follows that whenever the layperson’s interests conflict with those of the expert and the expert has high competence, i.e. when E is close to 1, then the layperson is only going to regret deferring to the expert in those cases where she was correct. It can be easily verified that the likelihood of this event ranges from 0.25 to 1, where the lowest chance is associated with L = E = 0.5.

For the case of conflicting interests, the probabilities for the other previously mentioned cases are given in Table 3.

**Table 3:** Conflict: probabilities of events

	Agreement	Disagreement
Correct expert advice	L · (1 − E)	(1 − L) · (1 − E)
Incorrect expert advice	(1 − L) · E	L · E

Let us briefly compare this table with Table 2, which concerned the probabilities of these cases under the assumption of mutual interests. The distinction between these tables is that the occurrences of E in Table 2 are replaced with (1 − E) in Table 3, and the occurrences of (1 − E) are replaced with E.

## Intermediate interest alignment and conditions for trust

Let us now proceed with the more complex case where the interests of the layperson and the expert neither fully align nor fully conflict. There will be many cases that do not fall neatly into either one of the two categories of scenarios that we’ve discussed so far. It is important to explore the possibility that the interests may sometimes conflict and sometimes align. To operationalize this idea, I propose to model *the degree of interest alignment* between the layperson and the expert by a probability α, where 0 ≤ α ≤ 1. One interpretation of α is that the probability that the interests align is α and the probability that the interests conflict is (1 − α). This probability can be understood in two distinct ways: (1) as the layperson’s credence in the proposition that their own interests align with the expert’s interests, or (2) as a more objective probability, reliability or robustness of their interest alignment. In any case, higher values for α are associated with higher degrees of interest alignment. I use this model to investigate the conditions under which one should trust and defer to experts; one of the main findings is:

(3.1) There are cases where laypeople should trust and defer to experts *even if* the interests of the layperson and the expert partially conflict.

To study trust, we will, once again, discuss the three events mentioned before. It seems plausible to think that the best possible circumstances arise when layperson’s competence, expert’s competence and interest alignment attain higher values. In the following, we investigate for each of the three previously mentioned events, how the likelihood of the event changes when the layperson’s competence, expert’s competence and/or interest alignment increases. Let me foreshadow the main qualitative findings regarding reducing the likelihood of the three events: (D) disagreement, (I) incorrect expert advice, and (R) layperson regrets deferring to the expert:

(3.2) Increasing the interest alignment will reduce the likelihood of all three events, irrespective of the competences of the layperson and the expert.
(3.3.) Increasing the competences of the layperson and/or the expert will reduce the likelihood of these events only in some circumstances.

I proceed with using the model to explain and substantiate these findings. First, what are the odds that the expert and the layperson disagree on which policy proposal to adopt? It is easy to verify that the likelihood of this event can be calculated by a convex combination of the previous two equations: (a) α times the likelihood of disagreement in the case of mutual interests plus (b) (1 − α) times the likelihood of disagreement in the case of conflicting interests. We give two equivalent equations:

$$P\left( D \right) = \alpha \cdot \left( {L \cdot \left( {1 - E} \right) + \left( {1 - L} \right) \cdot E} \right) + \left( {1 - \alpha } \right) \cdot \left( {L \cdot E + \left( {1 - L} \right) \cdot \left( {1 - E} \right)} \right),$$

$$P\left( D \right) = \alpha \cdot \beta + \left( {1 - \alpha } \right) \cdot \left( {1 - \beta } \right),$$

where β = L · (1 − E) + (1 − L) · E. In other words, β represents the probability that exactly one of the two agents succeeds in identifying the proposal that fits her own interests and, consequently, (1 − β) represents the chance that either both agents succeed in doing so or neither of them does. The second equation reformulates the first one using the abbreviation β. These equations illustrate that the likelihood of disagreement between the layperson and the expert (P(D)) depends on three variables: the layperson’s competence (L), the expert’s competence (E), and the degree of interest alignment (α).

Let us investigate how the likelihood of disagreement varies if we vary just one of the three variables (L, E, and α). To answer this question, consider the derivatives:

$$\frac{\partial P\left( D \right)}{{\partial L}} = \left( {1 - 2\alpha } \right) \cdot \left( {2E - 1} \right),$$

$$\frac{\partial P\left( D \right)}{{\partial E}} = \left( {1 - 2\alpha } \right) \cdot \left( {2L - 1} \right),$$

$$\frac{\partial P\left( D \right)}{{\partial \alpha }} = 2\beta - 1.$$

The first derivative shows how the likelihood of disagreement changes if we only change the layperson’s competence. Whether increasing the layperson’s competence increases or decreases the likelihood of disagreement depends on the sign of the first derivative.^19^ In other words, it depends on whether the expression (1 − 2α) · (2E − 1) is positive or negative. Given our assumption that E ≥ 0.5, we see that (2E − 1) ≥ 0. So, the sign of the expression is fully determined by the sign of (1 − 2α). Hence, the first derivative demonstrates that increasing the layperson’s competence will decrease the likelihood of disagreement *if and only if* the interest alignment exceeds 50%.

The second derivative shows how the likelihood of disagreement changes if we only change the expert’s competence. By similar reasoning, it follows that increasing the expert’s competence will decrease the likelihood of disagreement *if and only if* the interest alignment exceeds 50%.

The third derivative shows how the likelihood of disagreement varies if we only change the interest alignment. As mentioned in Sect. 2.1, 0.5 ≥ L · (1 − E) + (1 − L) · E = β and, therefore, 2β − 1 ≤ 0. Hence, the final equation illustrates that increasing the interest alignment will decrease the likelihood of disagreement, irrespective of the layperson’s and expert’s competence. Notice that the extent to which increasing the interest alignment will reduce the likelihood of disagreement does depend on the exact competences of the layperson and the expert: higher competences lead to greater reductions.

The second event discussed before is the one where the expert’s advice picks out the incorrect alternative (that is, the alternative that does not serve the layperson’s interests). The likelihood of this event is given by a convex combination of the previous two equations: (a) α times the likelihood that the expert is correct in the case of mutual interests plus (b) (1 − α) times the likelihood that the expert is correct in the case of conflicting interests. Hence:

$$P\left( I \right) = \alpha \cdot \left( {1 - E} \right) + \left( {1 - \alpha } \right) \cdot E.$$

Let us determine how the likelihood of incorrect expert advice varies if we change one of the two variables (E and α). Consider the derivatives:

$$\frac{\partial P\left( I \right)}{{\partial E}} = 1 - 2\alpha ,$$

$$\frac{\partial P\left( I \right)}{{\partial \alpha }} = 1 - 2E.$$

The first derivative illustrates that the effect of increasing the expert’s competence is not uniform: increasing the expert’s competence decreases the likelihood of incorrect expert advice *if and only if* the interest alignment exceeds 50%. Since 1 − 2E ≤ 0, the second derivative demonstrates that the correctness of the expert’s advice can be increased (and incorrectness decreased) by increasing the degree of interest alignment, irrespective of the expert’s competence. This should not come as a surprise.

The third pivotal event concerns the case where the layperson regrets having trusted the expert. Once again, the likelihood of this event is given by a convex combination of the previous two equations: (a) α times the likelihood of regret in the case of mutual interests plus (b) (1 − α) times the likelihood of regret in the case of conflicting interests. Hence:

$$P\left( R \right) = \alpha \cdot L \cdot \left( {1 - E} \right) + \left( {1 - \alpha } \right) \cdot L \cdot E.$$

To investigate the impact of each of the three parameters (α, L, and E) on the likelihood of regret, consider the following derivatives:

$$\frac{\partial P\left( R \right)}{{\partial L}} = \alpha \cdot \left( {1 - E} \right) + \left( {1 - \alpha } \right) \cdot E,$$

$$\frac{\partial P\left( R \right)}{{\partial E}} = L \cdot \left( {1 - 2\alpha } \right),$$

$$\frac{\partial P\left( R \right)}{{\partial \alpha }} = L \cdot \left( {1 - 2E} \right).$$

The first derivative illustrates that *decreasing* the layperson’s competence will decrease the likelihood of regret irrespective of the expert’s competence and the degree of interest alignment, since all the terms are positive. The second derivative demonstrates that increasing the expert’s competence decreases the likelihood of regret *if and only if* the degree of interest alignment exceeds 50%. Regarding the third derivative, notice that (1 − 2E) ≥ 0, since E ≥ 0.5. Hence, the third derivative illustrates that increasing the degree of interest alignment will reduce the likelihood of regret irrespective of the competences of the expert and the layperson. Notice that the role of the layperson’s competence and the degree of interest alignment is uniform, while that of the expert’s competence is not: increasing the expert’s competence reduces the likelihood of regret *only in some circumstances*.

Finally, let us characterize the conditions under which it is rational or advisable for the layperson to trust and defer to the expert. Let us assume that the layperson knows her own interests and, therefore, the likelihood that she herself finds out which alternative fits her own interests equals the value of her competence L. In comparison, the likelihood that the expert’s advice is in the interest of the layperson can be given by:

$$P\left( {\neg I} \right) = 1 - P\left( I \right).$$

Hence, under these assumptions, it is advisable to trust and defer to experts *if and only if* the following inequality obtains:^20^

$$L \le P\left( {\neg I} \right) = \alpha \cdot E + \left( {1 - \alpha } \right) \cdot \left( {1 - E} \right).$$

First, notice that it is advisable to trust and defer to experts *only if* the degree of interest alignment exceeds 50%. For suppose that the degree of interest alignment does not exceed 50%. Then, as indicated before, increasing the expert’s competence will increase the expert’s incorrectness and, therefore, decrease her correctness. Intuitively, this means that the expert is better able to identify the best option for the layperson but will advise against it since that option is not in the expert’s interest.^21^ It follows that a competent expert with misaligned interests is more problematic than an incompetent one. Under these circumstances, the best possible correctness of the expert obtains if her competence equals 50%. In that case, it holds that her correctness equals 50%. Hence, the expert’s correctness would not exceed 50% and would be lower than the layperson’s correctness. Therefore, if the degree of interest alignment does not exceed 50%, then it is surely not advisable to trust and defer to experts.

Second, let us proceed with considering the case where the degree of interest alignment exceeds 50%. As we saw before, increasing the expert’s competence will then decrease the expert’s incorrectness and, thus, increase her correctness. In the best possible scenario, the expert is fully competent and, therefore, her correctness equals the value of α. In particular, the expert’s correctness is at most α. Hence, if α ≤ L, then it is surely not advisable to trust and defer to experts. In other words, it is advisable to trust and defer to experts *only if* the degree of interest alignment exceeds the value of L.

Third, and finally, let us consider a numerical example to examine the conditions under which it is rational to trust and defer to experts. Consider the case where the layperson’s competence equals 60%. Table 4 depicts the conditions under which the expert’s correctness equals 0.6. Hence, this table can be viewed as depicting the conditions under which the expert’s correctness equals the layperson’s correctness. For instance, the column where E = 0.8 and α = 0.67 represents the fact that it is rational and advisable to trust and defer to an expert *if* the expert’s competence exceeds 80% and the degree of interest alignment exceeds 67%. As a result, laypeople should defer to experts *even if* the interests of the layperson and the expert do not fully align. More generally, given values for L and E, one should defer to experts in case α satisfies the following inequality^22^:

$$\frac{L + E - 1}{{2E - 1}} \le \alpha .$$

**Table 4:** The conditions under which expert correctness equals 60%: each column represents one possible scenario, represented by values for E and α

Expert competence (E)	0.6	0.65	0.7	0.75	0.80	0.85	0.9	0.95	1.0
Interest alignment (α)	1	0.83	0.75	0.70	0.67	0.64	0.63	0.61	0.60
Correct expert (P(¬I))	0.6	0.6	0.6	0.6	0.6	0.6	0.6	0.6	0.6

Once one accepts the idea that trust has two dimensions, some qualitative predictions of the model may not be surprising. The main innovation, therefore, is to bring both of these dimensions together in a simple model of trust. After all, the model explains that the rationality of trust intimately depends on the degree of interest alignment and on the levels of competence. Moreover, we have demonstrated that there exist cases where laypeople should trust and defer to experts *even if* the interests of the layperson and the expert partially conflict.

## Revising trust and indiscernibility

I have emphasized that under virtually any circumstance there is a risk that the layperson will regret having trusted the expert. This event may cause the layperson to refrain from trusting the expert in the future and thereby trigger a negative feedback loop. However, there are different ways in which one may update trust in view of such negative events. Typically, people are certain about neither the competence nor the interests of their partners in information exchanges. As a result, a negative event may be given at least three possible explanations:

- The other person is incompetent
- The other person has different interests
- The other person made an unlucky mistake

It may be hard to figure out which of these hypotheses is apt. After all, how do you distinguish an unlucky mistake from incompetence? It is tempting to think that observations about the expert’s judgements should allow the layperson to have a more accurate idea of the interests and competence of experts. This thought is, however, mistaken. Let me foreshadow the main findings of this section:

(4.1) It is logically impossible for laypeople to determine exactly the expert’s competence and the degree of interest alignment on the basis of the expert’s judgment and correctness.
(4.2) It is practically infeasible for laypeople to accurately determine the degree of interest alignment *even if* they know the expert’s competence.

Let us see why. For simplicity’s sake, let us consider the assumption that one always later finds out which policy proposal was in one’s interest. This means that the layperson for each piece of advice has several observable data points: her own judgment, the judgment of the expert, and which policy proposal fits her own interests (which is found out later). To demonstrate that these observable data do not enable the layperson to obtain an accurate idea of the sincerity and competence of the expert, it suffices to present two indistinguishable scenarios.

Let us use a simple numerical example to illustrate the point. In the first scenario, the expert is not very competent, but her interests fully align with those of the layperson. Let us say that E_1_ = 0.6 and α_1_ = 1.0. In the second scenario, the expert is very competent, but her interests do not fully align with those of the layperson. Let us say that E_2_ = 1.0 and α_2_ = 0.6. Can the layperson distinguish between these two scenarios based on the aforementioned observables? The only variation in the observable data would be the judgment of the expert. So, it is only possible for the layperson to distinguish between these scenarios if the likelihood of the expert’s correctness varies. However, as can easily be calculated using the equations from Sect. 3, in both cases the likelihood that the expert’s advice identifies the policy proposal that is in the layperson’s interest is 60%.

This observation can be generalized. Table 5 gives a small overview of some of the possible values for E and α and their associated likelihoods of expert’s correctness, regret, and disagreement.

Consider the scenario in Table 5 represented by values E = 0.9 and α = 0.63. The value for α in this column represents the degree of interest alignment for which an expert with competence 90% yields the same likelihood of expert correctness as an expert with competence 60% and interest alignment 100%. Hence, under these circumstances, it is *logically impossible* for the layperson to determine the expert’s competence and the degree of interest alignment on the basis of the likelihood of the expert’s correctness, the likelihood of regret and/or the likelihood of disagreement.

**Table 5:** Logical indiscernibility: each column represents one possible scenario, represented by values for E, L, and α

Expert competence (E)	0.6	0.65	0.7	0.75	0.80	0.85	0.9	0.95	1.0
Layperson competence (L)	0.6	0.6	0.6	0.6	0.6	0.6	0.6	0.6	0.6
Interest alignment (α)	1	0.83	0.75	0.70	0.67	0.64	0.63	0.61	0.60
Correct expert (P(¬I)	0.6	0.6	0.6	0.6	0.6	0.6	0.6	0.6	0.6
Regret (P(R))	0.24	0.24	0.24	0.24	0.24	0.24	0.24	0.24	0.24
Disagreement (P(D))	0.48	0.48	0.48	0.48	0.48	0.48	0.48	0.48	0.48

Notice that there are in fact more scenarios that are logically indiscernible based on the observable data; there are *infinitely many*. After all, it is easy to verify that any scenario where α · E + (1 − α) · (1 − E) = 0.6 cannot be distinguished based on the observable data.

I proceed with arguing that it is also *practically infeasible* to determine the exact degree of interest alignment and competence. The fundamental reason is statistical in nature: the observable data at most allow for the formulation of a *degree of confidence* in certain values, but do not allow for full certainty. The reason regarding logical indiscernibility above is more severe in that the given observable data do not allow the layperson to distinguish between several scenarios at all. There is no degree of confidence that distinguishes the scenarios discussed above.

Besides the fact that observable data cannot determine the competence and the benevolence of the expert with full certainty, there is the additional question of how confident one could determine the interest alignment *if we assume that the competence levels are known*. After all, one may possess independent evidence that a given expert is epistemically reliable in, say, 80 per cent of the cases. How much does the likelihood of previously mentioned events vary with the degree of interest alignment? Table 6 represents the simple numerical example where L = 0.6 and E = 0.8.

**Table 6:** Practical indiscernibility: each column represents one possible scenario, represented by values for E, L, and α

Expert competence (E)	0.8	0.8	0.8	0.8	0.8	0.8	0.8	0.8	0.8	0.8	0.8
Layperson competence (L)	0.6	0.6	0.6	0.6	0.6	0.6	0.6	0.6	0.6	0.6	0.6
Interest alignment (α)	0	0.1	0.2	0.3	0.4	0.5	0.6	0.7	0.8	0.9	1
Incorrect expert (P(I))	0.8	0.74	0.68	0.62	0.56	0.5	0.44	0.38	0.32	0.26	0.2
Regret (P(R))	0.48	0.44	0.41	0.37	0.34	0.3	0.26	0.23	0.20	0.16	0.12
Disagreement (P(D))	0.56	0.55	0.54	0.52	0.51	0.5	0.49	0.48	0.46	0.45	0.44

Table 6 demonstrates that it is extremely difficult to determine the interest alignment with accuracy. After all, it seems as though it will be hard to accumulate enough reliable observable data that would allow one to distinguish between an interest alignment of 0.6 and 0.8, because the associated likelihoods vary only mildly.

Let us expand the numerical example to demonstrate this. Suppose the layperson has competence 0.6 and the expert’s competence is known to be 0.8. Consider the case where the layperson gets ten pieces of advice from the expert and finds out that the expert was correct in seven out of these ten cases. It may be surprising that if the degree of interest alignment were 0.6, then there would be a 18% chance that the expert is correct in exactly seven out of ten cases, whereas this chance would be 26% if the degree of interest alignment were 0.8.^23^ Since these chances do not differ much, under these circumstances, one cannot confidently claim that the degree of interest alignment is 0.8 instead of 0.6.

In cases where the layperson cannot find out which policy proposal was in her interest, trust and distrust must be based on disagreements. However, Table 6 demonstrates that it seems virtually impossible to determine the degree of interest alignment based on the likelihood of disagreement; After all, the likelihood of disagreement varies from 56% (if α = 0) to 44% (if α = 1). As a consequence, it hard to imagine how one could rationally decide to distrust experts on the basis of disagreements.

## Conclusion: optimistic implications and outlook

To recap, let me illustrate how my model and analysis could apply to two cases that were mentioned in the introduction. Crucially, this is meant to show how my analysis *could* apply to a *simplified* example; the following is not meant to be a thorough or conclusive analysis of these real-world cases.^24^ First, consider a case where the general practitioner advises a particular treatment to a given patient. Should the patient trust and defer to the general practitioner? According to my analysis, it depends on the competences of the patient and the general practitioner, and the degree of interest alignment. Let us suppose that interests of the patient and the general practitioner align on 80% of the occasions, which implies that the degree of interest alignment can be set to 80%. Furthermore, given extensive university education and recurring training sessions, let us suppose that the general practitioner’s competence can be modelled by a probability of 90%. My simple model can then be used to demonstrate that the patient should trust and defer to the general practitioner if and only if the patient’s competence is below 74%.

Second, a policy expert may advise citizens to follow to a certain set of rules. Should the citizen trust and defer to the policy expert? It is plausible that the policy expert’s advice is aimed at serving the common interest irrespective of whether these fully align with the interests of this specific citizen. Although the policy expert does not try to determine the layperson’s interests, their interests may still align. Let us suppose that the layperson’s credence in the proposition that their own interests align with the expert’s interests is 70%. One reason for this overlap may be that the layperson’s own interests are partly based on the common interest. Furthermore, let us assume that the layperson does not have significant social scientific knowledge and, therefore, her competence can be modelled by a probability of 60%. My simple model can then be used to show that the citizen should trust and defer to the policy expert if and only if the policy expert’s competence exceeds 75%.

Let me proceed with some concluding thoughts. To summarize, in this paper I have argued for several pessimistic conclusions:

(5.1) Under some circumstances, it can be rational or advisable for laypeople not to trust and defer to experts (Sect. 2 and Sect. 3).
(5.2) It is logically impossible for laypeople to determine the expert’s competence and the degree of interest alignment only on the basis of the expert’s judgment and correctness (Sect. 4).
(5.3) It is practically infeasible for laypeople to accurately determine the degree of interest alignment even if they know the expert’s competence (Sect. 4).

So, is the conclusion that anything goes? No. What my model demonstrates is that even though the observable data do not allow laypeople to determine the exact competence of experts and the degree of interest alignment, they can rule out certain competence/interest-alignment pairs. For example, if a layperson observes that an expert’s judgment conforms to her interests in 9 out of 10 cases, then there is rational pressure to decrease the credence in the expert having fully conflicting interests, i.e. α = 0. Moreover, although laypeople often question both the competence and the sincerity of experts who express conflicting beliefs (see Kahan et al., 2011), the model entails that there are rational limits to questioning *both* the competence and the sincerity of experts.

The above considerations could be taken to indicate a relatively bleak picture where ordinary citizens can rationally refrain from deferring to scientists in questions concerning the underlying causes of climate change, the risks of non-vaccination, and the prospects of economic policy proposals. The model indeed supports the fact that there are circumstances where it is rational to refrain from deferring to experts. However, the model also specifies the particular conditions that need to be in place for laypeople to do so rationally (see the end of Sect. 3). In other words, the model can be used to specify the conditions under which citizens are rationally required to defer to experts; or to specify the conditions under which distrust in experts can thrive and pose risks to the implementation of important policies (e.g. conspiracy theories).

What are the conditions under which laypeople are rationally required to trust and defer to experts? Under the assumption that the expert is more knowledgeable than the layperson, closer inspection of the model reveals that the layperson can rationally refrain from deferring to the expert *only if* the expert’s interests substantially conflict with hers. Without any evidence of conflicting interests, it is thus plausible that the layperson is rationally obliged to defer to the expert. For example, Table 4 demonstrates that in the particular case where the layperson’s competence is 60% and the expert’s competence is 80%, it is rational for the layperson to refrain from deferring to the expert *only if* the degree of interest alignment is less than 0.67. Of course, the model predicts that this tipping point for the degree of interest alignment will be lower if the expert is more competent and/or if the layperson is less competent.

Moreover, the above reasoning holds in the particular case where the layperson is assumed to be able to accurately determine their own respective interests. It is plausible that they are not that perfect and, as a result, there will be more rational pressure to defer to experts than my simple model predicts.

Can a layperson determine the exact level of interest alignment *if* she knows the expert’s competence? I have noted that the prospects are relatively dim, especially for cases where the layperson does not find out the alternative that actually was in her personal interest. I’ve emphasized that it is implausible that a layperson can rationally and confidently distrust experts solely on the basis of disagreements (see Table 6). As a consequence, the prevalence of disagreements is not reliable evidence for a low degree of interest alignment.

It is interesting and important to investigate how distrust in experts can be prevented. My analysis highlights that a large part of successfully doing so would involve increasing the degree of interest alignment (or the perception thereof) rather than merely increasing the expert’s competence. The expert’s information may be flawless and her reasoning valid, but in the absence of sufficient interest alignment laypeople can still rationally refrain from deferring to experts. My analysis emphases two aspects of this: (1) Instead of interests that *fully* align, it suffices for the interests to align to a *sufficient degree*. Hence, to avoid distrust in experts, perfect mutuality is not needed. (2) Instead of interests that *actually* sufficiently align, to avoid distrust in experts it is important to influence laypeople’s *perception* of the degree of interest alignment.^25^

Let me end with a note on future work. Although I’ve been mostly concerned with the layperson’s individual decision regarding trust, it is clear that social factors and institutions play a significant role. In recent years, agent-based models of social influence (Flache et al., 2017) and information exchange (O’Connor and Weatherall, 2019; Stewart et al., 2019) have been used to study the spread of false and true beliefs in different social circumstances. Trust plays a central role in general social interactions (Cook et al., 2013), including information exchanges. The simple model of trust presented here (or an extension of it) can be used in such agent-based models to study the social factors that influence the spread of information in communities.

## Appendix to Sect. 3

As argued in the main text, one should trust and defer to experts if and only if the following inequality obtains:

$$L \le P\left( {\neg I} \right) = \alpha \cdot E + \left( {1 - \alpha } \right) \cdot \left( {1 - E} \right).$$

Notice that

$$\begin{aligned} P\left( {\neg I} \right) & = \alpha \cdot E + \left( {1 - \alpha } \right) \cdot \left( {1 - E} \right) \\ & = \alpha \cdot E - \alpha \cdot \left( {1 - E} \right) + 1 - E \\ & = \alpha \cdot \left( {2E - 1} \right) + 1 - E. \\ \end{aligned}$$

Hence, since 2E − 1 ≥ 0, the inequality can be rewritten as:

$$\frac{L + E - 1}{{2E - 1}} \le \alpha .$$

## Footnotes

1 An editorial in *The Lancet Infectious Diseases* concurs: “In the USA, the country is plagued by prescription opioid misuse fuelled by aggressive pharmaceutical marketing, the people of Flint, MI, have been without safe drinking water for 3 years, and the most basic drugs are often unaffordable because of profit-driven health care. Little wonder that some individuals question the authorities’ desire to prioritise their wellbeing” (‘Trust Issues’, 2019).

2 The epistemological question of whether testimony can give rise to knowledge is distinct (Lackey & Sosa, 2006 is a great source for the epistemology of testimony). On testimony and scientific knowledge, Hardwig (1991: p. 706) writes: “[M]uch scientific knowledge rests on the moral and epistemic character of scientists. Unavoidably so. Not because ‘hard data’ and logical arguments are not necessary, but because the relevant data and arguments are too extensive and too difficult to be had by any means other than testimony.”

3 In the literature on values in science, there is an ongoing debate about whether and how values influence scientists’ claims in cases of inductive risks. For instance, Douglas (2000: p. 565) writes: “The scientist will need to consider both the quantity of evidence or degree of confirmation to estimate the magnitude of inductive risk and the valuation of the consequences that would result from error to estimate the seriousness or desirability of the consequences. The weighing of these consequences, in combination with the perceived magnitude of the inductive risk (i.e. how likely one is to be wrong), determines which choice is more acceptable. Where inductive risks are involved and non-epistemic consequences follow from error, non-epistemic values are essential for deciding which inductive risks we should accept, or which choice we should make.” Of course, values may differ from interests and motivations; I therefore do not study the ramifications of my study for this debate on values in science.

4 This is in line with so-called motives-based accounts of trust. Two prominent such accounts are given by Hardin (2002) and Jones (1996), see McLeod (2015) for a more elaborate discussion of motives-based accounts.

5 Baier (1986: p. 235) concurs: “Where one depends on another's good will, one is necessarily vulnerable to the limits of that good will. One leaves others an opportunity to harm one when one trusts, and also shows one's confidence that they will not take it. Reasonable trust will require good grounds for such confidence in another's good will, or at least the absence of good grounds for expecting their ill will or indifference. Trust then, on this first approximation, is accepted vulnerability to another's possible but not expected ill will (or lack of good will) toward one.”

6 Trust may also play an important role in argumentative practices in that arguments could be dismissed when the source is distrusted—without engaging with its argumentative content (Dutilh Novaes, 2020).

7 Social exchange theorists agree that context plays a crucial role in trustworthiness (Mayer et al., 1995).

8 Both of these types are cases of *epistemic* trust since they concern trust in experts as providers of information.

9 Compare, Goldman (2001: p. 104), who considers the possibility that a sincere expert’s opinion may be inaccurate due to interests and biases: “Interests and biases can exert more subtle distorting influences on experts’ opinions, so that their opinions are less likely to be accurate even if sincere.”

10 The current COVID-19 crisis can provide telling examples. Often times, policy advice is geared towards averting health catastrophe and although this is also in the personal interest of individuals, laypeople may also care about the economic consequences and employment. One striking example where one might argue that the policy experts insufficiently considered the personal interests is the lockdown in India, which led to mass travelling (*The **Economist*, 2020).

11 The example on inductive risk and vaccination is inspired by the discussion of Irzik and Kurtulmus (2019: pp. 1161–1162).

12 Although I do not engage with the literature on values in science, my observation suggests that even if science cannot be value-neutral, this may not be problematic for low degrees of value misalignment.

13 Hardin’s (2002: pp. 4–5) encapsulated-interest account of trust may support this claim: “Note that *our merely having the same interests with respect to some matter does not meet the condition of trust as encapsulated interest*, although it can often give me reason to expect you to do what I would want you to do or what would serve my interests (because it simultaneously serves yours). The encapsulated-interest account does entail that the truster and the trusted have compatible interests over at least some matters, but such incentive compatibility, while necessary, is not sufficient for that account, which further requires that the trusted values the continuation of the relationship with the truster and has compatible interests at least in part for this reason.”

14 Hardin's (2002: p. 1) encapsulated-interest account of trust agrees that encapsulated interest is compatible with (partially) conflicting interests: “I trust you because I think it is in your interest to take my interests in the relevant matter seriously in the following sense: You value the continuation of our relationship, and you therefore have your own interests in taking my interests into account. That is, you encapsulate my interests in your own interests. My interests might come into conflict with other interests you have and that trump mine, and you might therefore not actually act in ways that fit my interests. Nevertheless, you at least have some interest in doing so.”

15 This notion of regret is widely used in psychology and behavioural economics (Bell, 1982; Bleichrodt & Wakker, 2015; Loomes & Sugden, 1982; Zeelenberg & Pieters, 2007). For example, Zeelenberg (1999) writes: “Regret is a negative, cognitively based emotion that we experience when realizing or imagining that our present situation would have been better, had we decided differently.” Accordingly, when we view the decision of the layperson as choosing between following her own judgment and deferring to the expert, then my treatment of regret aligns with this literature.

16 I follow a common practice from the literature on epistemic democracy and Condorcet Jury Theorems, where competence is often modelled using probabilities (see, for instance, Goodin & Spiekermann, 2018).

17 Of course, the assumption that laypeople are more competent than chance level may not be met in all domains. For example, Brennan (2016, Ch. 2) argues that laypeople are worse than chance level in the political domain. All in all, one could easily drop this assumption from my analysis and the model, and investigate the ramifications for trust.

18 The following may be helpful for remembering these notational conventions: A_0_ is in the layperson’s interest if the truth value of Q is 0, and A_1_ is in the layperson’s interest if the truth value of Q is 1.

19 For our present purposes, the sign of a number x can be specified as follows: the sign of x is negative if x < 0 and positive if x ≥ 0.

20 It may be interesting to note that this holds even if one would drop the assumption that the layperson’s competence is higher than chance level. In addition, the following second and third observation would apply to such cases.

21 Recall that the expert may actually be motivated by altruistic considerations, yet fail to identify the layperson’s interests. That is, the expert need not be egoistic. In such a case, the layperson should not trust and defer to the expert even though the expert is benevolent.

22 See “Appendix A” for a derivation of this fact.

23 These chances can be calculated using binomial distributions. In general, under these circumstances, the chance that an expert with degree of interest alignment α is correct in seven out of ten cases is given by the binomial distribution with parameters k = 7, n = 10, p = α ⋅ E + (1 − α) ⋅ (1 − E) = 0.6α + 0.2.

24 Simply stated, according to my analysis whether a specific layperson should trust and defer to a specific expert depends on the exact competences of that layperson and that expert, and on their exact degree of interest alignment. Determining these competences and the degree of interest alignment requires empirical calibration.

25 Of course, this observation cuts both ways: distrust in experts may be the result of a perception of conflicting interests while the interests actually align.
